# Supplementary material for: Parkin contributes to synaptic vesicle autophagy in Bassoon-deficient mice
Source: eLife. 2020 May 4;9:e56590. doi: 10.7554/eLife.56590 (PMC7224700; doi:10.7554/eLife.56590)
Supplement: Figure 3—source data 1. — Detailed mass spectrometry information on ubiquitinated peptides depicted in Figure 3. This table includes the full name of the protein, the ubiquitination site(s) within the peptide (in amino acids), the quantification of the peptides (log2) for both WT and Bsn KO neurons, the fold increase in ubiquitinated peptides and the Mascot and Andromeda scores. [file elife-56590-fig3-data1.docx]

| Abbre-viation | Protein | Ubiquiti-nation site(s) | WT (log2) | Bsn KO (log2) | fold increase | Mascot/  Androme-da score |
| --- | --- | --- | --- | --- | --- | --- |
| E2N | Ubiquitin conjugating enzyme E2N | 92 | 23.38 | 26.36 | 7.89 | 24/100 |
| E2N | Ubiquitin conjugating enzyme E2N | 94 | 21.04 | 24.36 | 9.97 | 50/126 |
| GAPDH | Glyceraldehyde 3-phosphate dehydrogenase | 111 | 24.10 | 22.50 | 0.33 | 76/141 |
| GAPDH | Glyceraldehyde 3-phosphate dehydrogenase | 213 | 22.00 | 22.70 | 1.59 | 56/79 |
| Nrx1 | Neurexin1 | 1493 | 20.40 | 20.80 | 1.34 | 26/76 |
| B3A3 | Anion exchange protein | 642 | 22.04 | 21.67 | 0.77 | 61/109 |
| SNAP25 | Synaptosomal nerve-associated protein 25 | 76 | 19.40 | 21.40 | 4.07 | 42/70 |
| Stx1b | Syntaxin 1b | 55 | 22.00 | 22.90 | 1.94 | 48/86 |
| Stx1b | Syntaxin 1b | 69 | 22.30 | 23.80 | 2.76 | 46/213 |
| Stx1b | Syntaxin 1b | 93 | 20.50 | 21.60 | 2.17 | 47/85 |
| Stx1b | Syntaxin 1b | 71 | 21.40 | 23.20 | 3.38 | 57/97 |
| Stx1b | Syntaxin 1b | 188 | 22.90 | 24.10 | 2.28 | 90/172 |
| SV2a | Synaptic vesicle protein 2a | 143 | 19.50 | 22.80 | 9.49 | 30/97 |
| SV2b | Synaptic vesicle protein 2b | 333 | 19.80 | 22.20 | 5.19 | 24/44 |
| SV2b | Synaptic vesicle protein 2b | 341 | 21.40 | 22.30 | 1.85 | 27/98 |
| SynGAP | Ras/Rap GTPase activating protein | 1127 | 20.31 | 20.59 | 1.22 | 65/90 |
| Syt1 | Synaptotagmin1 | 119 | 19.60 | 21.60 | 4.04 | 39/67 |
| Syt1 | Synaptotagmin1 | 133 | 19.70 | 21.00 | 2.49 | 33/62 |
| Syt11 | Synaptotagmin11 | 72 | 18.90 | 22.70 | 14.00 | 22/49 |
| Syt11 | Synaptotagmin11 | 124 | 19.80 | 20.60 | 1.71 | 29/59 |
| Syt5 | Synaptotagmin5 | 62 | 22.70 | 23.40 | 1.58 | 110/232 |
| Tuba1b | Tubulin alpha-1B | 163 | 22.90 | 22.54 | 0.78 | 78/77 |
| Vamp2 | Vesicle-associated membrane protein 2 | 59 | 22.40 | 22.60 | 1.15 | 46/118 |
| Vamp2 | Vesicle-associated membrane protein 2 | 52 | 22.90 | 23.60 | 1.69 | 59/109 |
| V-ATPase E1 | V-type proton ATPase subunit E1 | 10 | 17.87 | 20.66 | 6.94 | 62/130 |
| VGlut1 | Vesicular glutamate transporter 1 | 272 | 21.40 | 21.60 | 1.23 | 67/160 |
